# Supplementary material for: The prevalence of M. tuberculosis among acid fast bacilli cultures from military health system and veterans affairs beneficiaries in Hawaii and the Pacific Islands from 2002 to 2019
Source: PLoS One. 2021 Jul 9;16(7):e0254312. doi: 10.1371/journal.pone.0254312 (PMC8270159; doi:10.1371/journal.pone.0254312)
Supplement: Attachment — Submitted filename: Response to reviewers.docx [file pone.0254312.s002.docx]

**Review Rebuttal Responses regarding:**

The prevalence of *M. tuberculosis* among acid fast bacilli cultures from military health system and Veterans Affairs beneficiaries in Hawaii and the Pacific Islands from 2002 to 2019

We appreciate the constructive feedback provided by both reviewers and the opportunity to revise our work in hopes of publication. Below, we address each comment received.

**Reviewer 1:**

1. Lack of inclusion of NTM results.
   a. We did gather the data on the NTM-positive AFBs and plan to publish those results separately.

**Reviewer 2:**

Overall, big picture messages:

1. VA beneficiaries are not MHS beneficiaries. I understand that TAMC has an agreement with the VA and also care for VA beneficiaries, but they are not the same and VA is not typically included in MHS population denominators for rates.
   a. We have separated the results of MHS and VA beneficiaries.
2. Need to ensure that analysis is of individuals rather than specimens because of the necessary assumption of independence. Report tests & positive tests per subject and explain policies & procedures which led to these findings.
   a. Changed results and analyses to be by individual.
3. Prevalence is not incidence or an incidence rate. I recommend obtaining population at risk and calculating actual rates among MHS beneficiaries. This would mean eliminating VA, RC, NG, and PIHCP patients from calculations of rates, as you won’t have denominators for these groups.
   a. Changed calculations to be prevalence based on number of MHS and VA enrollees over the study period, respectively.
   b. Included Reserve Corps and National Guard individuals in results and analyses since those RC and NG individuals included in our study are those who were on ‘active’ status at time of sample collection.
   c. Removed PIHCP individuals from the analysis as they are a separate demographic group.
4. Recommend further investigation of potential outbreak in 2009 through discussion with public health, including obtaining genotype data to assess previously unsuspected transmission and outbreaks.
   a. The authors have elected to review this data separately and potentially publish a review of all cases of MTB in this sample, Of note, there appears to be no link between any of the cases and no known outbreak of TB during this time.
5. Recommend shifting discussion from force health protection impact, which is not demonstrated, to testing of low risk populations, which is suggested by these data.
   a. Removed detailed discussion of FHP from discussion section.

Specific comments:

1. Abstract line 29: how does this prevalence of 1% compare to other laboratories in Hawaii, CDC, or in the military? Is it higher or lower? This should be part of the comparison to the literature in the discussion section. I hypothesize that this is a low prevalence of positive cultures but am not sure, which is why comparison data are needed. Then you can further hypothesize why any differences identified exist—likely due to unique policies on TB testing of certain patient types at the Tripler Hospital. I suspect over-testing of low risk populations based on the data provided.
   a. Included comparison to Hawaii and United States
2. Line 32: “low rate” –evidence for this is not discussed in the abstract—Prevalence is not a rate. I agree that you should try to ascertain rates so you can make valid comparisons. It is not clear from the data in this manuscript that force health protection measures are effective or making any impact whatsoever. Actual rates of disease would be more compelling.
   a. Discussion now in case rates
3. Line 40 intro: “most” should be quantified. 70% of all US cases are non-US born. Is this higher on Hawaii?
   a. Over 80% born in Asia or US-affiliated Pacific Islands
4. Line 46: MAJOR Problem (potentially) is the inclusion of a different population: VA beneficiaries. These are NOT the same population as MHS beneficiaries and should be excluded from the analysis. This is in contrast with MHS retiree beneficiaries, which is NOT the same thing.
   a. Addressed as above
5. Lines 55-57: Not clear what is meant by “unknown true positive rate” or what is being signified here
   a. Removed
6. Line 58-59: “high risk” overstates the case. US Service members are a lower risk than the general population, but TB may occasionally occur deployment. Recommend you revise this statement
   a. Revised to ‘higher’ risk
7. Line 62: reference 9 is the incorrect reference—should be 8. Please ensure all references are correctly matched. Strongly recommend use of endnote or some other similar software to avoid mistakes like this.
   a. This has been corrected.
8. Line 63: “stringent” probably overstates the case, as does “high risk”. Please revise to accurately convey that screening is meant to test only those at high risk and discourage testing among those at low risk—which is most of the US military population.
   a. Revised
9. Line 71-73: Please revise to state something like “…evaluates the prevalence of TB among those MHS beneficiaries who were tested for TB at Tripler Army Medical Center...”
   a. Revised
10. Methods line 84: which reference laboratory was used? Hawaii? What were the results? (how did TAMC compare with the reference lab?)
    a. Samples were isolated and preliminary identification was performed at TAMC, thereafter samples had to be sent to reference laboratories for further testing. The reference laboratory varied over the study period and was dependent on whichever lab had the contract at the time. That information was not deemed pertinent for inclusion in the study.
11. Lines 89-90: Real problems with identification of MHS beneficiaries evident here. If you’re including NG, then you should also have Reserve Component as well. As above, VA should be considered separately from MHS, as it is completely distinct and separate population. VA are not typically considered part of the MHS beneficiary population (in contrast, retirees who enroll in MHS care are), nor are NG/reserves. I would probably present VA, NG, and RC completely separately as none are typically counted as MHS beneficiaries—what should be the denominator when calculating rates.
    a. Addressed.
12. Lines 99-111: MAJOR issue is the fact that multiple samples should be present for each individual, which means the analysis presented is invalid since independence is an assumption of regression analysis. If you have multiple samples per subject, then your assumption of independence is not met, and your analysis is not valid. I would expect 3 samples for each person who did not have TB (since it takes 3 specimens to rule out TB among TB suspects), and I would expect more for those who did have TB—at least 5 (additional specimens after completion of 2 months of therapy and to test for cure after 5-6 months). This should be clarified and the analysis revised to include only individuals rather than specimens, or some other method of accounting for this dependence. Also the number of specimens per individual should be presented. If it isn’t close to the numbers above, then a detailed explanation of what the policies are at TAMC and why they differ from the established standard of care.
    a. Follow-up for treatment of tuberculosis and subsequent AFB sample collection is performed by Hawaii Department of Health. So samples obtained during initial diagnosis/hospitalization were obtained within our system, however follow-up samples are not available in our system.
13. Results Line 115: as above, it doesn’t sound like these are individuals, but rather samples/specimens, and this should be presented as individuals. We need to know how many unique individuals had TB out of how many unique individuals were tested. As supplemental information, please describe the number of tests and number of positive tests, including distributions of these numbers per person.
    a. Revised to present results by individuals
14. Line 121: data are not typically “stratified” by the outcome, but rather by exposures. Just say when comparing cases and non-cases or something similar.
    a. Revised
15. Table 1: use row percents, not column percents—your outcome is prevalence and the rows give that. Column percents are more difficult to interpret. Also, where are VA beneficiaries in this table? Recommend separating out active component as a specific group, and presenting both RC and NG as a separate MHS beneficiary group.
    a. Revised
16. Line 128: why was Fisher’s exact test used for sex specifically? Not clear—this is typically used for cell sizes <5, so I would have expected any of the other variables.
    a. Fisher’s exact test is designed to assess unadjusted associations for 2x2 contingency tables, which is why it was used for gender and not the other variables.  We used chi-squared tests to evaluate associations for higher order m x n contingency tables, i.e., for variables with more than two levels.
17. Line 131 and 135—these numbers are highly suspect to me, since they indicate that >90% of TB suspects had only one sample, when the standard of care is at least 3. Please clarify and explain in detail the policies and procedures at TAMC and how and why they deviate from standard US practice.
    a. Addressed.
18. Line 142: The larger number of cases in 2009 is worth further examination and investigation. Was this an outbreak? Were these cases identified during contact investigations? Please contact TAMC public health to get further information on whether an outbreak occurred. With their assistance, you can also contact HI state public health department to get genotyping information to look for evidence of transmission and outbreaks which were not identified during contact investigations.
    a. This information is outside the scope of this paper, however could be addressed as part of the follow-up publication.
19. Table 2: Recommend presentation of unadjusted odds ratios as well as a separate column. This will bring into sharp focus the higher prevalence of the PIHCP population; it is not surprising that this association is not significant in adjusted analysis due to collinearity with Asian/PI race/ethnic group.
    a. PIHCP population removed
20. Also you should present the number of observations used in the adjusted analysis so that we can evaluate the impact of missing data. I suspect that around half of the data were used in this analysis?
    a. The adjusted analysis used data from all subjects. Race/ethnicity was the only factor with missing data, but instead of excluding subjects with missing race from the analysis we included a separate category of other/unknown.  Of 1283 subjects classified as other/unknown race, 927 were reported as other, 274 were reported as null, 76 were reported as unknown, and 6 were reported as Western Hemisphere Indian.
21. Why did you combine Dependent/retiree/civilian in this table, which is different from table 1? I recommend doing it the same way as table 1.
    a. Civilians are no longer included. Because there were only 4 MHS retirees with a positive TB test, and positivity rates of TB did not differ significantly between retirees and dependents those groups were combined to maximize power and simplify the comparison to be active duty vs. others.
22. Discussion line 163: MAJOR recommendation—get population at risk and calculate rates, and present this as part of the analysis in the RESULTS section. I like that you have the overall, but you shouldn’t drop it in at the last minute in the discussion section. Presentation of rates (probably you won’t be able to do more than unadjusted rates) overall, by year, and by demographic groups should be possible either by going through TRICARE or by putting in a request to AFHSD. This would complement your analysis by providing compelling data which is not subject to the biases introduced by arbitrary hospital procedures for TB testing. It is also much easier to compare to other state, and military rates. That should go in the results section, and then you can discuss further in the discussion section. However, you will need to think carefully about which groups can be included in your analysis: you will likely have to exclude VA, RC, and NG (and the Pacific Partnership patients) when calculating rates, as they are not typically included in the denominators of MHS beneficiaries. Anyone not included in the denominator should be excluded from the numerator.
    a. Revised.
23. Your discussion section should follow standard format: key results, comparison to the literature, strengths/limitations, judicious interpretation of significance of findings. It should not start with new data not presented in the results.
    a. Revised
24. Line 165: “Overestimate”—this should be clarified. If you are including non-MHS beneficiaries in your case counts, then you will get an overestimate of your rates since they are not in the population at risk (denominator). If you exclude those from your analysis of rates (as I recommend), then you’ll have a slight underestimate since you may miss some MHS beneficiary cases which are diagnosed and treated outside of the MHS.
    a. Revised
25. Line 175-185: I’m skeptical about these conclusions about FHP. It is not clear that anything mentioned here has had an impact on FHP. In fact, your data suggests that there may have been an unrecognized outbreak in 2009! You also show a higher prevalence of positive cultures in this finding, which is quite odd and inconsistent with good FHP. I suspect when you look at rates, the rates among AD will actually be lower. I recommend that you frame this through the lens of screening procedures at TAMC and how and why they are testing patients there, which I suspect will explain a lot of the differences seen in prevalence of positive cultures. My suspicion is that there is some policy or accepted procedure which results in lots of testing of low risk individuals, particularly retirees.
    a. Addressed.
26. Line 183: I don’t think the evidence supports these statements of “increased risk”—recommend softening this to acknowledge that all available data suggests that military personnel are actually at lower risk.
    a. Revised to clarify that we are comparing them to non-traveling persons in the U.S.
27. Line 186: “on the island of Oahu” – not sure where this comes from, and this is the first time this is mentioned. I recommend just sticking with previous language of MHS beneficiaries. Also, don’t use “rates” unless you actually use rates in your analysis—prevalence doesn’t have a time component so it cannot be a rate.
    a. Revised
28. Line 188-190: As above, recommend that you expand upon these unusual assessment procedures and how they may impact your data and the conclusion you draw from them.
    a. Revised.
29. Line 192-3: “effective mitigation strategy” –I’m not convinced that your data indicate or support this statement. I’m more struck that it shows what happens when you overtest a low prevalence population—you get a lot of negatives.
    a. Revised
30. Lines 194-205. I don’t find the discussion of homelessness particularly enlightening, and it is highly speculative. Please provide supporting data which show that MHS beneficiaries are at significant risk for homelessness. It may be relevant for VA beneficiaries, but again this is a distinct population which should be analyzed completely distinctly.
    a. Removed
31. Lines 216-7: please assess and acknowledge the effect of collinearity on PIHCP estimates
    a. PIHCP removed from study group
32. Line 223: I think this manuscript would have more value if it attempted to elucidate these reasons through examination of policies and procedures, discussion with Public Health regarding contact investigations and outbreaks (including genotyping), and calculation of rates.
    a. This information may be considered for future publications, however was not within the initial intention of this publication.
33. Line 227: How many of the 11 cases in 2009 were AD? This may have been driving the TB rates in the overall military in 2009.
    a. 1 of 7 were active duty. We have included this information in the revised manuscript.
34. Line 240: Typically much more of an issue in dependents rather than military service members, please clarify.
