# Supplementary material for: The prevalence of M. tuberculosis among acid fast bacilli cultures from military health system and veterans affairs beneficiaries in Hawaii and the Pacific Islands from 2002 to 2019
Source: PLoS One. 2021 Jul 9;16(7):e0254312. doi: 10.1371/journal.pone.0254312 (PMC8270159; doi:10.1371/journal.pone.0254312)
Supplement: Attachment — Submitted filename: Response to reviewer.docx [file pone.0254312.s003.docx]

**Review Rebuttal Responses regarding:**

The prevalence of *M. tuberculosis* among acid fast bacilli cultures from Military Health System and Veterans Affairs beneficiaries in Hawaii and the Pacific Islands from 2002 to 2019

We appreciate the constructive feedback provided by the reviewer and the opportunity to revise our work in hopes of publication. Below, we address each comment received.

1. Running title probably would better reflect the content as “M. tb among military & veteran beneficiaries in Hawaii” or something similar

- Edited

2. Abstract line 30: MHS and VA beneficiaries “who were tested” or something similar

- Edited

3. Intro line 56-61: Typically populations are presented in the results section and belong there, and how you obtained them are in the methods section.

- Edited

4. Methods section: missing a description of how the population was obtained—ie. describe policies are in place to obtain the specimens. They should be all TB suspects, but please clarify this and any special, additional policies and procedures in place which may lead to additional specimens here. I’m thinking of the “screening” procedures listed later in the manuscript, which need to be clearly articulated. It sounds like this is primarily routine testing with a TST or IGRA on arrival, which would involve clinical evaluation of all positives and thus likely lead to more low-risk specimen collection based on symptom endorsement. Also should be clarified here who gets these screening procedures—all arrivals, all military, only medical personnel, only hospital assigned personnel, etc, as that is not clear.

- Added lines 85-87 to clarify how these samples were submitted to lab.
- These were not screening tuberculosis samples, they were all clinical specimens submitted ordered by physicians. The presumption is that all of these were submitted based on ATS/IDSA/CDC guidelines. To our knowledge none of these samples were submitted during research screening testing.

5. Line 118: military status is variously described as duty status and patient category in the manuscript. Please pick one and stick with it for consistency.

- Changed to military status

6. Line 121: case rates should be described as incidence rates, as that is the standard terminology, throughout the manuscript.

- Changed to incidence rates

7. Line 122: As above, need to describe data source for populations (military and VA), who obtained from, etc.

- Included in methods (lines 104-106 and 108-110)

8. Table 1: recommend you call these Total MHS and VA beneficiaries “tested for AFB”

- Edited

9. Line 159-161: I’m not sure that the comparisons of rates were done correctly—these are calculated differently than standard 2X2 tables. Please confirm you are using comparisons for rates, for example a simple and easy to use one is available at [www.openepi.com](http://www.openepi.com) Please also clarify which population this comparison refers to.

- Edited, please see lines 170-175

10. Figures 1, 1A and 1B; these are confusing. Figure 1 is fine but is not referenced in the body of the paper that I can find. 1A and 1B are the same figures in the package I received—this needs to be corrected. You could also replace these with a side by side bar graph which makes the comparisons between VA and military easier. Either way, the denominators of person-time used for both military and VA calculations should be explicit somewhere in the manuscript, either below the graph like in Figure 1 or elsewhere.

- The Figure 1 you are referencing was from the initial submission and is not intended for inclusion in the revised manuscript. The file inventory appears to have all prior submissions regardless of intended inclusion in the revision.
- The revised submission had no Figure 1, but had Figure 1A and Figure 1B. Based on your feedback for Figures 1A and 1B, they were consolidated into a new Figure 1 (side-by-side bar graph).
- Added a details regarding how the annual and cumulative incidence rates were calculated in the methods section (lines 127-130). Person-years were not used to calculate the rates. We used the population numbers by year and over the entire study period instead of cumulative person-time as the denominator for our incidence calculations.
- The numerical ranges of enrolled MHS and VA beneficiaries now explicitly stated in lines 161-164.

11. Discussion. Nice and helpful discussion of rates. However, as the title of the manuscript is prevalence among AFB cultures, and most of the analysis focuses on this, please at least add some comments on the key findings of that part of the analysis. In particular, it is worth commenting on the 1% prevalence of positive cultures, whether that was expected, how it compares to other studies, etc.

- Please see lines 155-156 in results and 218-220 in discussion

12. Line 219-221: this assertion needs to be backed up in the results section by some evidence, such as epi investigations or genotyping, then you can interpret it here if you have that. It is not clear what you’re basing this assertion on. Another explanation for the 2009 bump is random variability and small sample sizes.

- Edited, please see lines 239-240

13. Line 224-228: As above, this needs to be better supported in the methods section with information on local screening policies and procedures. Then you can discuss what the differences are which may affect your results. What about other difference in selection of population—i.e. more Native Hawaiian and Asian population which has a known higher rate of TB. What about increased travel to TB endemic areas in Asia and PI?

- There are many theories that we discussed as being possible causes for the differences in our results, however due to the lack of clear cause of the differences this section was removed.

14. Line 235-8: “MHS dependents…” as above, please describe these policies and procedures in the methods section.

- Removed this section.

15. Line 238-9: “Our observations…” This is a dubious claim which doesn’t have much support from the data—again, I’m still unclear what that screening program consists of. I recommend softening it to something more neutral, or stating that further work can be done to evaluate the effectiveness of the screening program.

- Removed this section.

16. Line 241: awkward wording: I suggest “ universal health care coverage” or something similar

- Removed this section.

17. Line 241-247” as in comment 15 above, I’m not really convinced about the effect of the screening program. My interpretation is that you have higher rates than most military populations but lower rates than the local/state populations. Your interpretation of the effect of screening should at least take this into account. Overtesting of the MHS population would be expected to result in higher incidence rates by increased, active case finding. In contrast, it would result in a lower proportion of positive specimens—ie. prevalence among AFB specimens.

- Removed this section.

18. Limitations line 263 and after: to me the most important is selection bias, both in the screening procedures which may have tested low risk suspects, and in the potential for biased numerators and denominators among VA beneficiaries. I disagree with the assessment that the number of VA beneficiaries receiving their care outside military facilities is small (line 270-1)—please provide substantiating data that this is the case in Hawaii, or at least in the VA population in general. All my prior experience with the VA population suggests that this is not the case. If you don’t have data to support, I would characterize the uncertainty in these statement. Similarly, I find the statement in 279-281 dubious, at least for the VA population—I expect that most cases are actually captured elsewhere.

- Edited; deleted prior lines 279-281. Addressed previous questions regarding screening procedures; no additional screening procedures using AFB testing are in place.

19. Line 266: minor point, but this is really 18 years or 17+.

- Edited

20. Line 285-6: as above, I think the incidence fits nicely within the context of a higher rate in the local population and a lower rate in the US military population – it is in the middle, which is really what you would expect.

- Edited wording
